# Exome-wide survey of the Siberian Caucasian population

**DOI:** 10.1186/s12881-019-0772-4

**Published:** 2019-04-09

**Authors:** Andrey A. Yurchenko, Nikolai S. Yudin, Mikhail I. Voevoda

**Affiliations:** 1 The Federal Research Center Institute of Cytology and Genetics, The Siberian Branch of the Russian Academy of Sciences, Lavrentieva 10 St, Novosibirsk, Russia 630090; 2 Novosibirsk State University, Novosibirsk, 630090 Russia; 3 Institute of Internal and Preventive Medicine-branch of Institute of Cytology and Genetics, Siberian Branch of Russian Academy of Sciences, 175/1, B. Bogatkov Street, 630089 Novosibirsk, Russia

**Keywords:** Exome sequencing, Population structure, Associations, Siberia, Russia

## Abstract

**Background:**

Population structure is an important factor in the genetic association studies but often remains underexplored for many human populations. We identified exome variants in 39 Siberian Caucasian individuals from Novosibirsk, Russia and compared their genetic allele frequencies with European populations from 1000 Genomes Project.

**Methods:**

The study participants were from Novosibirsk and represented people with monogenic diabetes, healthy individuals and a cohort from the tick-borne encephalitis study. Isolated DNA was enriched using Agilent SureSelect V5 kit and sequenced on Illumina HiSeq 4000 and genetic variants were identified using GATK pipeline. To estimate the patterns of the population structure we used PCA and ADMIXTURE analysis. Pharmocogenetically and medically important variants were annotated based on PharmGKB and ClinVar databases.

**Results:**

The analysis identified low, but highly significant population differentiation attributed to numerous loci between the Siberian Caucasian population and other European population samples as well as a higher proportion of the Finnish genetic component in the studied sample. The medical and pharmacogenetic annotation of highly significantly differentiated variants between the Novosibirsk and the combined European populations revealed a number of important genetic polymorphisms located in such genes as *FCGR3B*, *TYR*, *OCA2*, *FABP1*, *CHEK2* and *SLC4A1*.

**Conclusions:**

The study reports for the first time an exome-wide comparison of a population from Russia with European samples and emphasizes the importance of population studies with medical annotation of variants.

## Background

Population structure is a very important factor in medical genetic association studies which can compromise modern genomic methods not being properly accounted for. In Russia, population studies were mainly conducted using Y-chromosome or mitochondrial markers with the recent application of microarray methods [1–3] and did not allow to estimate the functional role of variants. Some recent phylogeographic studies used whole-genome sequencing with samples from Russia to elucidate history of migrations in Eurasia, but used small samples from diverse populations [4, 5]. In this study, we identified exome genetic variants for 39 individuals from Novosibirsk, Russia and compared them with the previously published genome-wide data and exomes of European populations from the 1000 Genomes Project to understand the level of the exome-wide divergence and the extent of the population stratification. The Novosibirsk population (NVSB) is of particular interest because it exhibits an example of a modern big city population affected by political and economic events of the twentieth century which changed the historical landscape of ethnic diversity of the former USSR territory through increasing urbanization, mass migration across the country and rapid demographic growth. In this study, we identified exome genetic variants for 39 individuals from Novosibirsk, Russia and compared them with the previously published genome-wide data and exomes of European populations from the 1000 Genomes Project to understand the level of the exome-wide divergence and the extent of the population stratification. Additionally, we tested allele frequency differences between our sample and combined European dataset for medically and pharmacogenetically important variants to identify loci which can be important for national studies.

## Methods

The study participants (*n* = 39) were from Novosibirsk and represented people with monogenic diabetes (*n* = 10), healthy individuals (*n* = 7) and a cohort from the tick-borne encephalitis study (*n* = 22). The participants signed an informed consent and defined themselves as ethnic Russians. The ethnicity of the participants was additionally checked prior the analysis with data from 1000 Genomes Project and two samples identified as clear outliers (close to the Asian populations) were excluded from the analysis. Isolated DNA was enriched using Agilent SureSelect V5 kit in and sequenced on Illumina HiSeq 4000 with 150PE reads. After the quality control with Trimmomatic [6] the reads were aligned with BWA mem [7] to Hg19 reference genome and processed with SAMtools [8]. Single nucleotide variants (SNVs) and indels were identified using GATK [9] according to the GATK Best Practices workflow for germline variation with the sensitivity filter equal to 99.9. The resulted VCF file was combined with 1000 Genomes Project genotypes [10] using bcftools [11] *merge* and filtered with VCFtools [11] at maximum 10 missed genotypes (*−-max-missing-count*) keeping only biallelic sites.

We performed the analysis on the two levels: with the Finnish (FIN) population for population genetic analysis (PCA, ADMIXTURE, Fst) and without FIN population to test the allele frequency differences for clinically and pharmacogenetically important variants. The FIN population was excluded from the second analysis as the most divergent European population with unique history [12]. To reduce the influence of the tightly linked loci on the patterns of population structure we applied the linkage-disequilibrium pruning using PLINK V1.93 software (Table 1). To estimate the patterns of the population structure we used the Principal Component Analysis (PCA) realised in SNPrelate [13] with European (1000 Genomes Project) and previously published Russian Siberian populations [2]. The proportions of genetic ancestry between populations were estimated using ADMIXTURE [14] for K = 2–8 (Table 1) and tested using Cross Validation Error estimation (CVE). To estimate and test statistically the level of pairwise population differentiation (Fst, [15]) we used *smartpca* software of the EIGENSTRAT package [16].

**Table 1:** Number of variants and filters applied to them for various analysis

Analysis	Number of variants	Filters
PCA	5948	LD < 0.3, only autosomes, MAF = 0.05, LD window = 100kbp
ADMIXTURE	55,669	LD < 0.2, only autosomes, LD window = 50kbp
Fst (*smartpca*)	65,436	only autosomes, MAF = 0.05
Allele frequency difference	117,010 (SNV) 5989(INDEL)	PLINK: --assoc fisher-midp mperm = 1,000,000

We annotated the variants using ANNOVAR [17] and PharmGKB [18] databases and then tested medically (ClinVar, [19]) and pharmacogenetically relevant variants for the differences in allele frequencies between the NVSB population and the combined non-Finnish European (NFE) dataset with PLINK v1.93 [20] using 1 M permutations.

The average coverage of the studied exomes varied from 47.7X to 71.3X. In total, we identified 136,276 SNVs and 14,464 indels in the studied dataset. Merging with data from 1000 Genomes Project produced a dataset of overlapped variants consisted of 117,010 SNVs and 5989 indels.

## Results

**Fig. 1:**
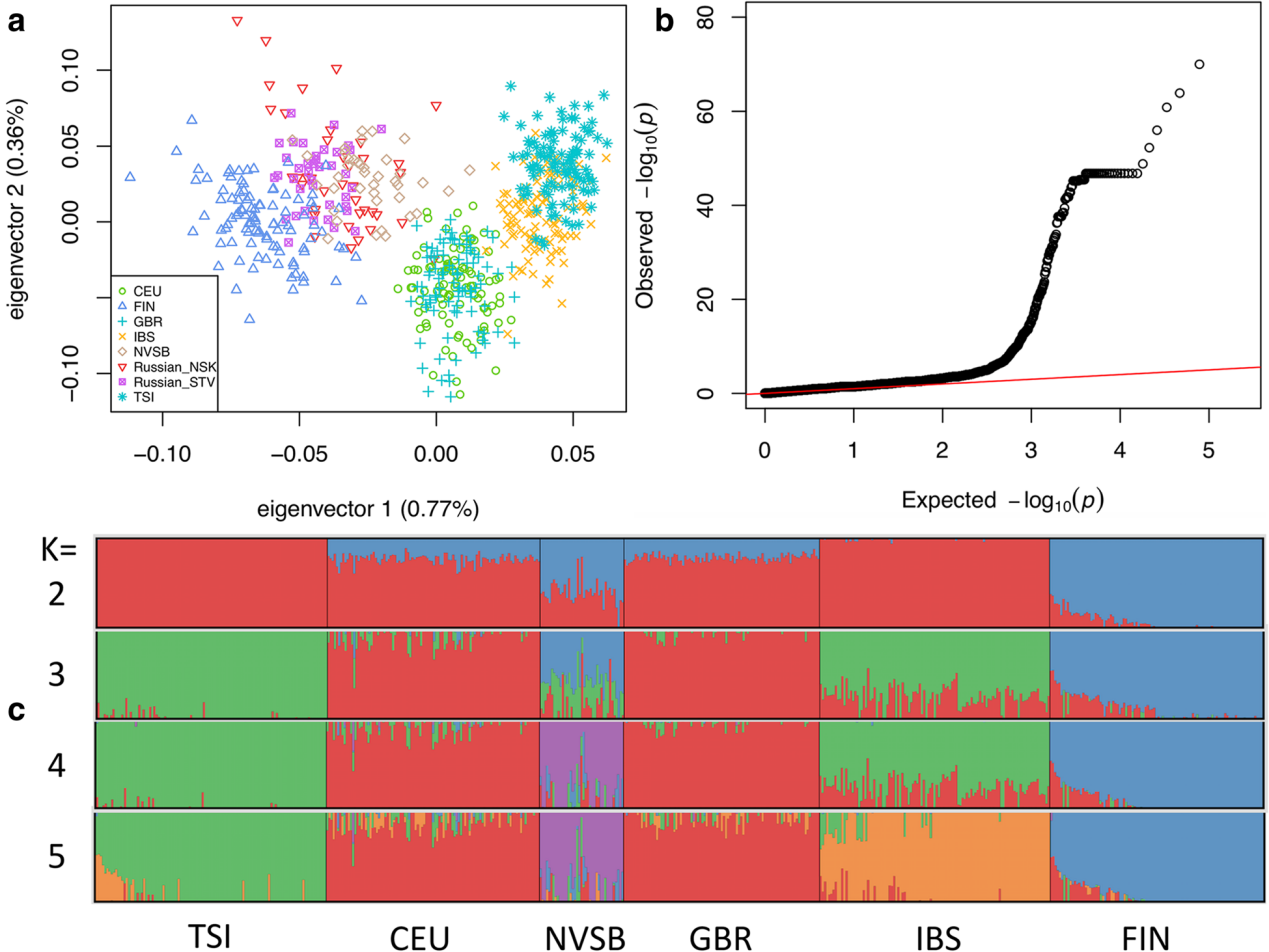
**a** Principal Component Analysis (Russian_NSK and Russian_STV are Russian from Novosibirsk and Siberian starovers respectively from [2]) **b** Observed and expected *P*-value distribution for allele frequency differences between NVSB and combined NFE sample **c** Results of the ADMIXTURE analysis for K = 2–5

During the population genetic analysis, the first principal component accounted for 0.77% of the total variation and separated all the populations (Fig. 1a) except closely related American (CEU) and British (GBR). The second principal component accounted for 0.36% of the total variation and separated mostly Tuscan (TSI) and Spanish (IBS) samples. Novosibirsk population (NVSB) was placed between the Finnish (FIN) and CEU with GBR samples and was clearly distinguished from them. The Russian Siberian populations from a previous microarray-based study [2], represented by a similar Caucasian Siberian population (Russian_NSK) and partially isolated Siberian Starovers (Old Believers, Russian_STV) were not distinguished between each other and samples from our study (NVSB).

In ADMIXTURE analysis, the lowest value of the Cross Validation Error was attributed to the K = 2, which captured the divergence of FIN from other European populations. NVSB demonstrated a higher proportion of the ancestral Finnish-related genetic component at K = 2 and at K = 3 relative to other populations. A new cluster (green) consisted of TSI and IBS appeared at K = 3 and then at an additional ancestral component emerged (K = 4) clearly separating NVSB (Fig. 1c, purple). Lastly, at K = 5, the IBS was separated from the rest of the samples.

The pairwise Fst values between all the populations except the CEU and GBR (*P*-value = 0.048) were highly significant (*P*-value < 1.1656e-11) albeit low (Fst = 0.002–0.013). The NVSB population demonstrated the highest level of differentiation with TSI (Fst = 0.009) and the lowest with GBR and CEU (Fst = 0.005). The results of the test for allele frequency differences between NVSB and NFE populations demonstrated pervasive inflation of the *P*-values attributed to numerous loci (Fig. 1b).

Among the 452 pharmocogenetically and 210 medically important variants we found 3 and 7 variants respectively (Table 2) which showed significant allele frequency differences between the NVSB and NFE population after the multiple testing correction (BH adjusted *P*-value < 0.05). The most significant differences in allele frequencies were attributed to such genes as *FCGR3B*, *TYR*, *OCA2*, *FABP1* and *SLC4A1* genes.

**Table 2:** Genetic variants from PharmGKB (*P*-value < 0.01) and ClinVar (BH adjusted *P*-value < 0.05) databases which demonstrated highly significant differences in allele frequency between NVSB and NFE

Chr:Position	SNP ID	Reference allele/subsitution	Frequency in NVSB	Frequency in NFE	*P*-Value	BH adjusted *P*-value	Gene	Drug (PharmGKB evidence level)/Clinvar annotation (number of reports)	Phenotype	
2:88424066	rs2241883	T/C	0.5513	0.3171	3.55E-05	0.016046	FABP1	fenofibrate (3)	Hypertriglyceridemia	PharmGKB variants
14:64700045	rs944050	T/C	0.141	0.03682	0.0002365	0.047987333	ESR2	gemcitabine (3)	Pancreatic Neoplasms	
15:75129594	rs2290573	G/A	0.2949	0.5095	0.0003185	0.047987333	ULK3	imatinib (3)	Leukemia, Myelogenous, Chronic, BCR-ABL Positive	
5:79950508	rs1105525	C/T	0.2949	0.1425	0.001166	0.131758	DHFR	methotrexate (3)	Precursor Cell Lymphoblastic Leukemia-Lymphoma	
1:161479745	rs1801274	A/G	0.3205	0.5071	0.002335	0.211084	FCGR2A	trastuzumab (2B)	Breast Neoplasms	
1:70904800	rs1021737	G/T	0.3974	0.247	0.005383	0.2702056	CTH	busulfan, cyclophosphamide (3)	Hemopoietic stem cell transplant	
1:171076966	rs2266782	G/A	0.5256	0.3587	0.004475	0.2702056	FMO3	sulindac, itopride (3)	NA	
1:230845794	rs699	A/G	0.5769	0.4097	0.005978	0.2702056	AGT	atenolol, irbesartan (3)	Hypertension	
2:65296798	rs7572857	G/A	0.3077	0.1746	0.00565	0.2702056	CEP68	aspirin (3)	Asthma	
11:126162843	rs8177374	C/T	0.07692	0.1983	0.00425	0.2702056	TIRAP	ustekinumab (3)	Psoriasis	
3:113890815	rs6280	C/T	0.8077	0.6615	0.008071	0.307736667	DRD3	risperidone (3)	Autistic disorder	
7:150696111	rs1799983	T/G	0.7821	0.6306	0.00817	0.307736667	NOS3	cyclophosphamide, doxorubicin, fluorouracil, methotrexate (3)	Breast Neoplasms	
1:161599693	rs448740	T/C	0.97436	0.6627	1.00E-06	0.00021	FCGR3B	Pathogenic (1)	Neutrophil-specific antigens na1/na2	ClinVar variants
11:89017961	rs1126809	G/A	0.0641	0.272	4.00E-06	0.00042	TYR	Benign(2);Likely benign(1); Pathogenic(3); Uncertain significance(1)	Albinism, melanoma	
15:28228553	rs74653330	C/T	0.0641	0.001188	9.00E-06	0.00063	OCA2	Likely benign(2);Pathogenic(1); Uncertain significance(1)	Tyrosinase-positive oculocutaneous albinism	
1:145507765	rs201779890	G/C	0.05128	0.001188	0.0001065	0.0037275	RBM8A	Pathogenic/Likely pathogenic​	Radial aplasia-thrombocytopenia syndrome	
17:42338993	rs45562031	C/T	0.1154	0.02138	8.30E-05	0.0037275	SLC4A1	Likely benign(2);Likely pathogenic(1); Pathogenic(1); Uncertain significance(2)	Spherocytosis type 4	
22:29121087	rs17879961	A/G	0.05128	0.001188	0.0001025	0.0037275	CHEK2	Likely pathogenic(4);Pathogenic(7); Uncertain significance(2)	Cancer of multiple types, susceptibility	
16:89986144	rs1805008	C/T	0.1667	0.06176	0.001211	0.03633	MC1R	Likely benign(2);Pathogenic(1)	Skin conditions	

## Discussion

In this study, we used an exome-wide dataset for the first time to study the population structure of the Caucasian Siberian population from a big Russian city Novosibirsk. The exome-wide survey of the Novosibirsk population demonstrated its genetic congruence with the previously published Russian dataset including the partially isolated Siberian Starovers regardless of the dramatic migration and demographic changes of the previous century. The Caucasian Novosibirsk population is quite homogeneous (Fig. 1a) and significantly differentiated from other European populations from 1000 Genomes Project demonstrating a relatively higher Finnish component which is presumably ancestral but not a result of recent migrations according to the ADMIXTURE results (Fig. 1c). This genetic differentiation although low in absolute Fst values should be taken into account during association studies. We identified 10 medically relevant SNVs with statistically significant allele differences between the NVSB and NFE populations including rs2241883 in* FABP1* gene previously associated with polycystic syndrome [21] and toxicity of fenofibrate [22], rs1801274 variant in *FCGR2A* gene shown to be important for the efficiency of trastuzumab in breast neoplasms [23], the rare rs17879961 variant in *CHEK2* gene reliably associated with predisposition to breast and colorectal cancer [24] and showed elevated frequency in NVSB. These variants should be studied in future on an expanded dataset with associated clinical data.

## Conclusion

The study reports for the first time an exome-wide comparison of a population from Russia with European samples and emphasizes the importance of population studies with medical annotation of variants.
